# Supplementary material for: Brain insulin resistance impairs hippocampal synaptic plasticity and memory by increasing GluA1 palmitoylation through FoxO3a
Source: Nat Commun. 2017 Dec 8;8:2009. doi: 10.1038/s41467-017-02221-9 (PMC5722929; doi:10.1038/s41467-017-02221-9)
Supplement: Supplementary file 1 — Supplementary Information [file 41467_2017_2221_MOESM1_ESM.pdf]

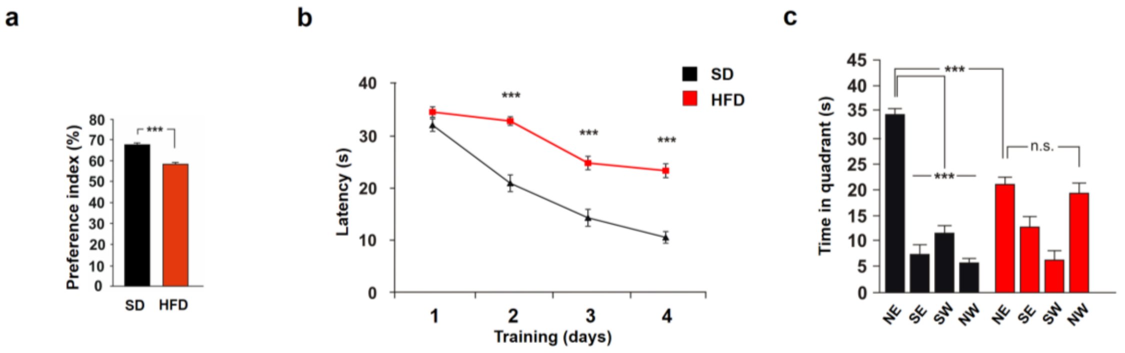

Supplementary Figure 1. HFD impairs learning and memory. **(a)** Preference for the novel object in NOR paradigm. **(b)** Latency to reach the hidden platform of SD and HFD mice in MWM. **(c)** Time spent in the four quadrants during probe test performed on day 5 of MWM test. In particular, NE (North-East) is the quadrant where the platform was placed during the training (target quadrant). (n = 8 for each group; statistics by unpaired Student's *t*-test). ***$p < 0.001$; n.s., not significant.

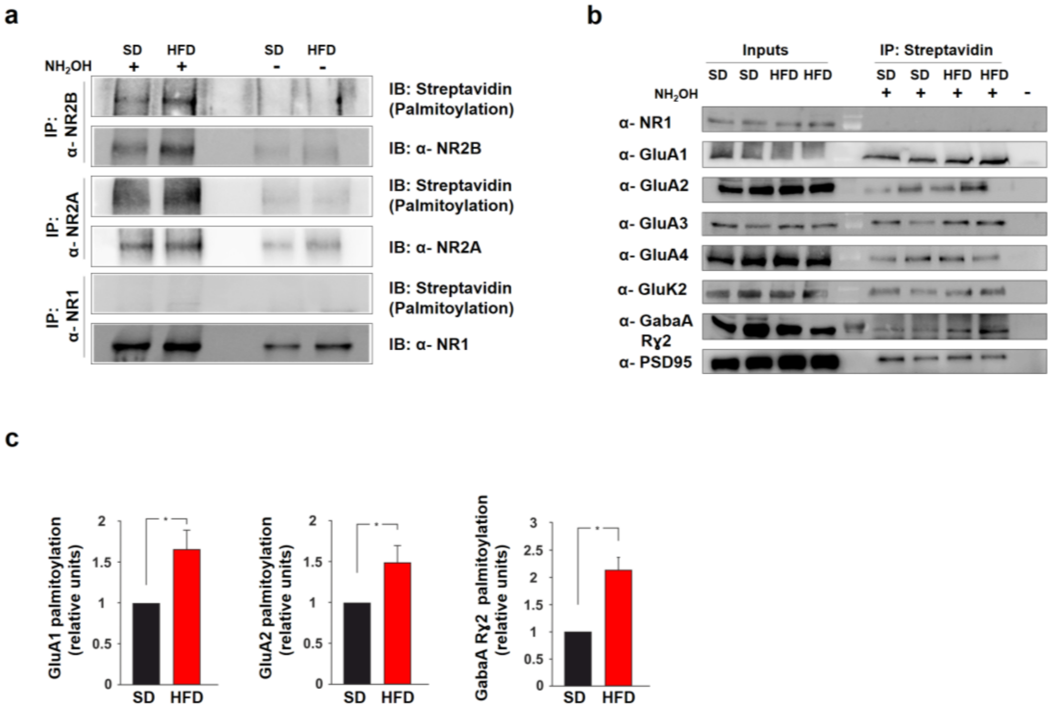

Supplementary Figure 2. Impact of HFD on palmitoylation of the main glutamate receptor subunits. (a) Palmitoylation of endogenous NR2B, NR2A and NR1 was examined in the hippocampus of SD and HFD mice using a biotin switch assay for palmitoylation. Palmitoylated (Acyl-Biotinyl Exchanged and detected by Streptavidin) protein fraction (top) and total immuno-precipitated protein (bottom) for each NMDAR subunit between SD and HFD mice. Samples without NH_2OH are negative controls. The experiment was repeated 3 times with 3 different tissues per group. **(b)** Representative Western blot showing palmitoylation (Streptavidin pull down-palmitoylation also named "proteomic ABE", see Methods) of the glutamate receptor subunits known to be palmitoylated and the main zDHHC3 targets (i.e. PSD95, GabaA Rɣ2) in the hippocampus of SD and HFD mice. Samples without NH_2OH are negative controls. **(c)** Densitometry of palmitoylated GluA1, GluA2 and GabaA receptor ɣ2 was normalized to total protein (inputs) expression (n = 4; statistics by Mann-Whitney test). Data are expressed as mean ± SEM. *$p < 0.05$.

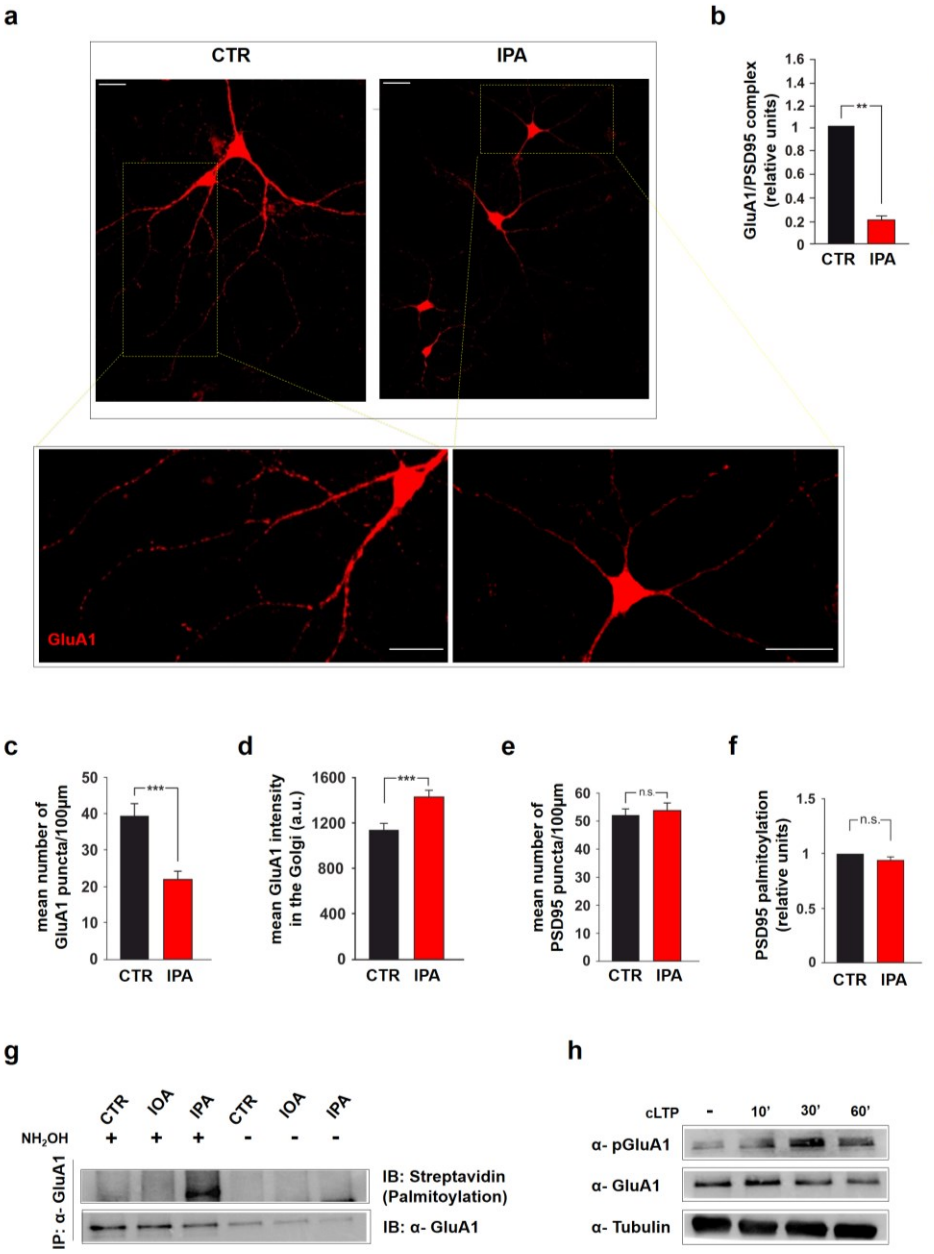

Supplementary Figure 3. IPA inhibits GluA1 trafficking to the synaptic membrane. **(a)** Confocal images of immunofluorescence staining for GluA1 (red) in the soma and neurites of hippocampal neurons upon IPA treatment. In each panel a magnification of GluA1 staining in neurites is shown in the box (bottom). Scale bar: 25 µm. **(b)** Quantified data of the ratio of GluA1/PSD95 (left) and GluA1/Actin (right) interaction showed in Figure 4d. PSD95 or Actin bound to GluA1 was normalized to both GluA1 amount in the immunocomplex and each protein amount in the total lysate. The experiment was repeated 4 times (statistics by Mann-Whitney test). **(c)** Mean number of GluA1 puncta (per 100 µm dendritic length) in neurons treated with vehicle (CTR, n = 54 dendrite segments) or IPA (n = 56 dendrite segments) for 24 hours (statistics by unpaired Student's *t*-test). **(d)** Mean intensity of GluR1 fluorescence in the Golgi apparatus (statistics by unpaired Student's *t*-test). **(e)** Mean number of PSD95 puncta (per 100 µm dendritic length) in hippocampal neurons (dendrite segments: CTR, n = 69; IPA, n = 70; statistics by unpaired Student's *t*-test). **(f)** Densitometry of palmitoylated PSD95/total protein (analyzed by Streptavidin pull down-palmitoylation also named "proteomic ABE", see Methods) upon IPA treatment (n = 3; statistics by Mann-Whitney test). **(g)** Palmitoylation of GluA1 was examined (by ABE assay) in hippocampal neurons upon treatment with vehicle, IOA or IPA. Palmitoylated GluA1 (top) and total immuno-precipitated protein (bottom). Samples without NH_2OH are negative controls. Experiment was repeated 3 times with similar results. **(h)** pGluA1 Ser^{845} in hippocampal neurons upon cLTP (containing in mM: 0.3 glycine, 0.03 picrotoxin, 0.01 strychnine and 0.001 tetrodotoxin) time course application. Experiment was repeated 3 times with similar results. Data are expressed as mean ± SEM. $^{**}p < 0.01$; $^{***}p < 0.001$; n.s., not significant.

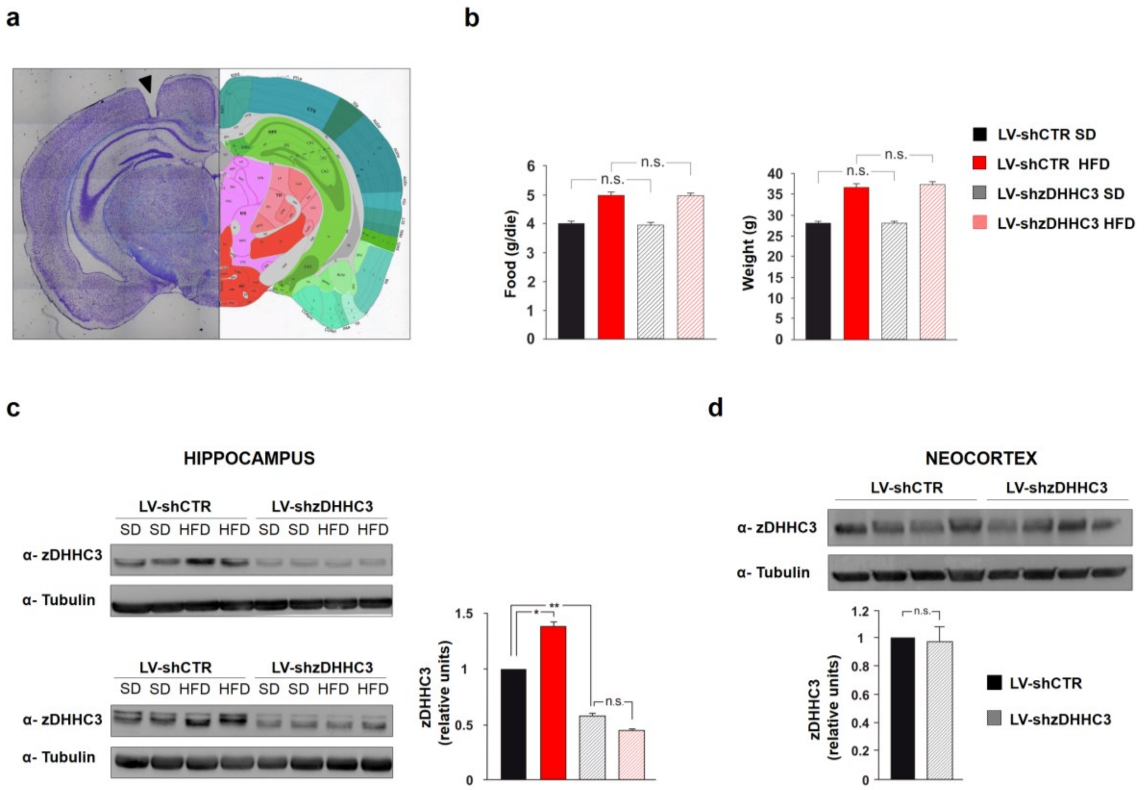

Supplementary Figure 4. Lentiviral injection was specifically performed in the hippocampus of mice. (a) Images showing cannula tracks and needle-tip placement in the hippocampus. The brain was Nissl-stained. Image credit: Allen Institute (http://mouse.brain-map.org/static/atlas; Coronal - image 79)[1]. **(b)** Food consumption (left) and weight (right) of mice fed with SD or HFD and injected with lentiviral particles harboring control shRNA (LV-shCTR) or shRNA against zDHHC3 (LV-shzDHHC3) (n = 8 per each group; statistics by two-way ANOVA and Bonferroni post-hoc). **(c)** Representative Western blots analysis of zDHHC3 expression in hippocampi of LV-shCTR and LV-shzDHHC3 mice. On the right, densitometry of zDHHC3 expression normalized to Tubulin in the hippocampus of LV-shCTR SD (black bar), LV-shCTR HFD (red bar), LV-shzDHHC3 SD (bar with black stripes) and LV-shzDHHC3 HFD (bar with red stripes) mice (n = 8 per each group; $F_{3.07} = 28.97$, LV-shCTR SD vs. LV-shCTR HFD $p = 0.014$; LV-shCTR SD vs. LV-shzDHHC3 SD $p = 0.001$; LV-shzDHHC3 SD vs. LV-shzDHHC3 HFD $p = 0.15$; statistics by two-way ANOVA and Bonferroni post-hoc). **(d)** Representative Western blots analysis of zDHHC3 expression in neocortex from LV-shCTR and LV-shzDHHC3 mice. On bottom, densitometry of zDHHC3 expression normalized to Tubulin (n = 8 per each group; statistics by unpaired Student's *t*-test). $*p < 0.05$; $**p < 0.01$; n.s. not significant.

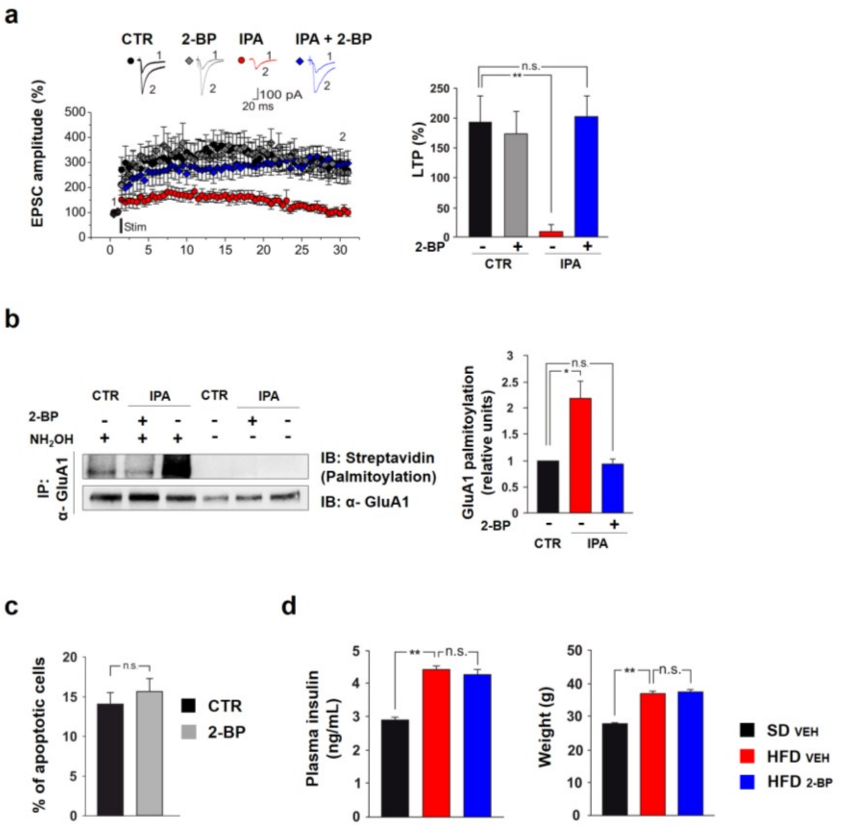

Supplementary Figure 5. 2-BP does not affect synaptic plasticity, neuronal survival and peripheral metabolism. **(a)** Time course (left) of LTP at CA3-CA1 synapses in rat hippocampal organotypic slices treated with vehicle (CTR, n = 12 slices), the palmitoylation inhibitor 2-bromopalmitate (2-BP, n = 10 slices), IPA (n = 11 slices) or IPA + 2-BP (n = 10 slices) for 24 hours. Results are expressed as percentages of baseline EPSC amplitude (= 100%). Insets (top) show representative EPSC at baseline (1) and during the last 5 min of LTP recording (2). Bar graphs (right) comparing mean LTP values during the last 5 min in the above mentioned conditions (194.0 ± 44.7%, CTR; 172.9 ± 37.5%, 2-BP; 8.9 ± 14.0%, IPA; 202.0 ± 37.6%, IPA + 2-BP; $F_{2,96} = 5.97$, CTR vs. IPA $p = 0.007$; CTR vs. IPA + 2-BP $p = 0.55$; statistics by two-way ANOVA and Bonferroni post-hoc). **(b)** Representative Western blots of palmitoylated GluA1 (top) and total immuno-precipitated protein (bottom) in rat hippocampal organotypic slices upon treatment with vehicle, IPA or IPA + 2-BP (5 µM). Samples without NH_2OH are negative controls. On the right, densitometry of palmitoylated GluA1/total immuno-precipitated GluA1 amount ratio (n = 3; statistics by Mann-Whitney test). **(c)** Bar graphs showing the percentage of apoptotic neurons upon the treatment with 5 µM 2-BP for 24 hours (statistics by unpaired Student's *t*-test). **(d)** HFD_{VEH} and $HFD_{2\text{-}BP}$ mice show not significant differences for plasma insulin levels (left) and weight (right) after treatments. The ELISA assay was performed in duplicate (n = 10 mice per group; statistics by one-way ANOVA). Data are shown as mean ± SEM. *$p < 0.05$; **$p < 0.01$; n.s. not significant.

Figure 1 d

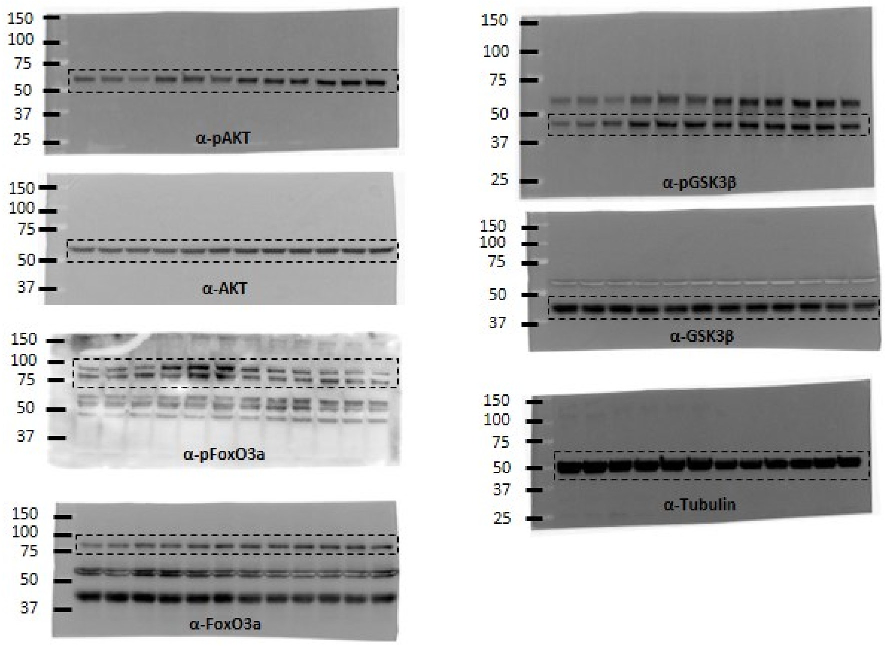

Supplementary Figure 6. The uncropped version of gel images presented in Figure 1.

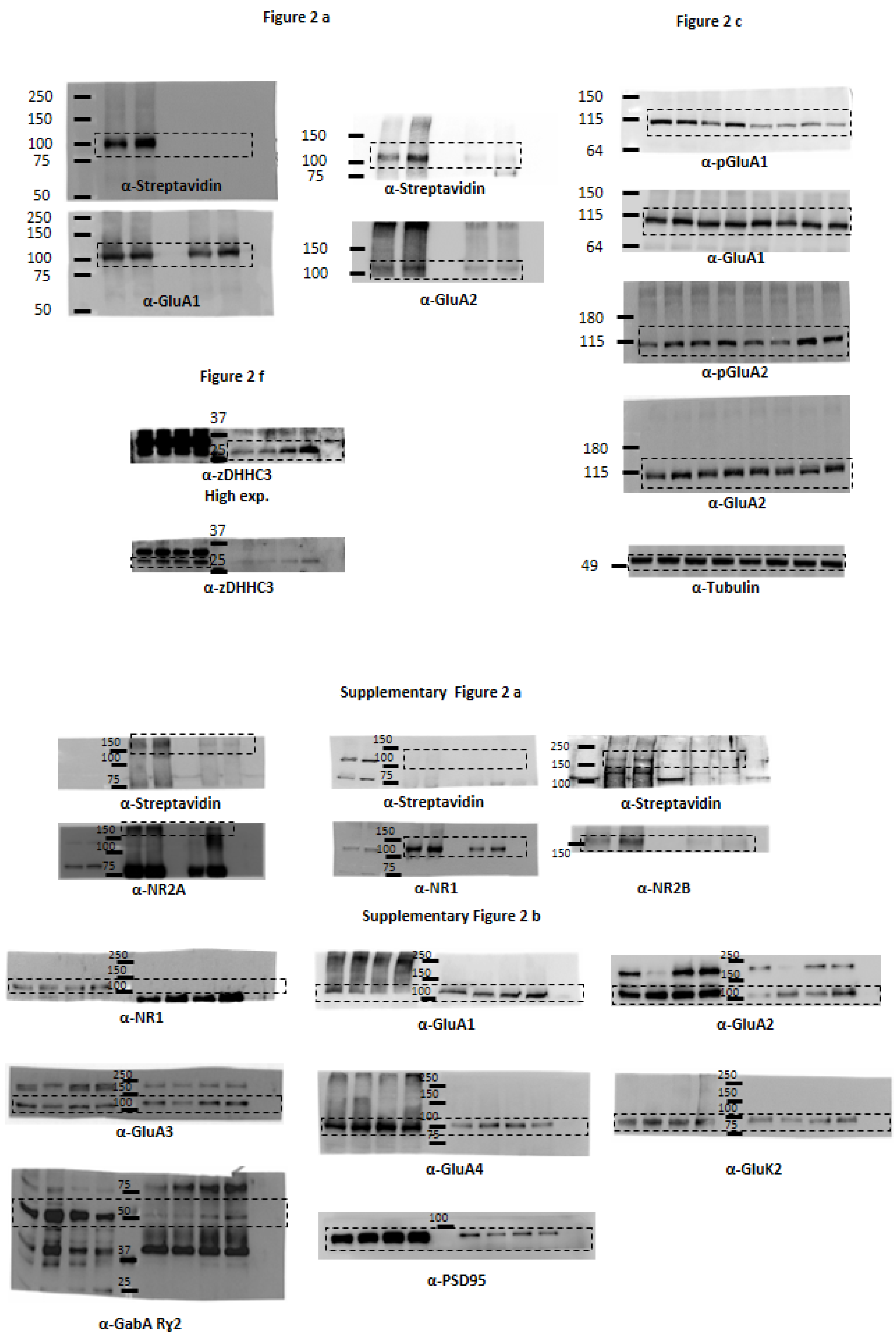

Supplementary Figure 7. The uncropped version of gel images presented in Figure 2 and Supplementary Figure 2.

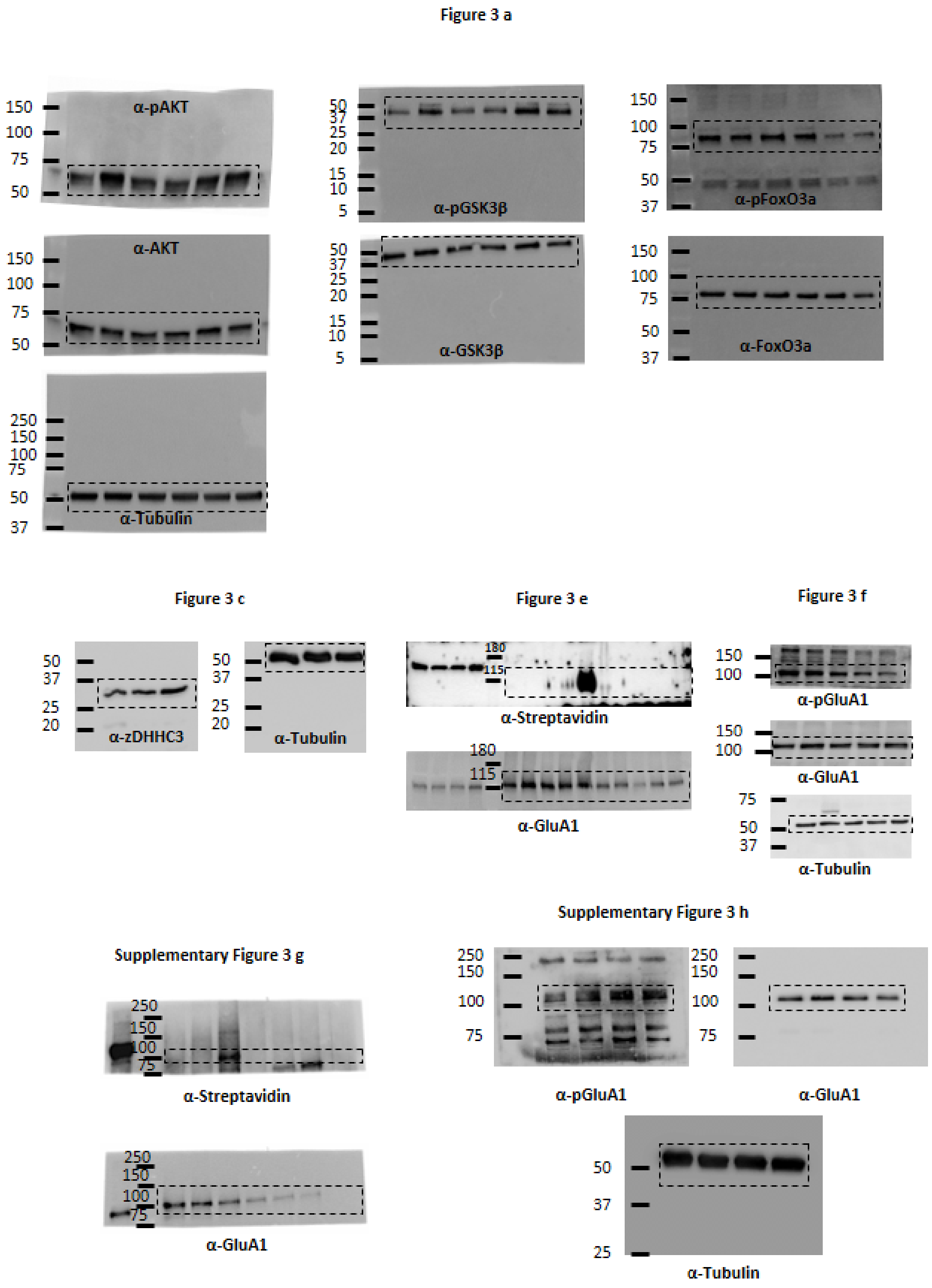

Supplementary Figure 8. The uncropped version of gel images presented in Figure 3 and Supplementary Figure 3.

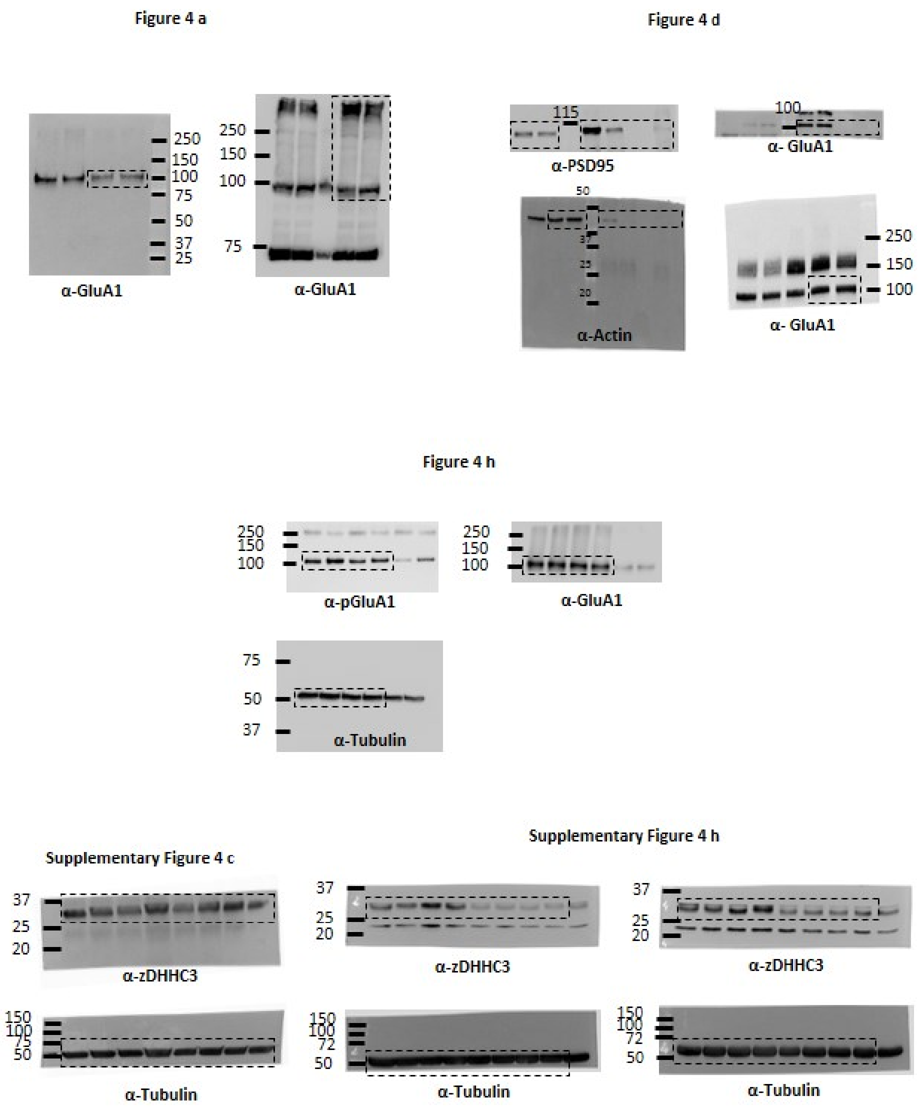

Supplementary Figure 9. The uncropped version of gel images presented in Figure 4 and Supplementary Figure 4.

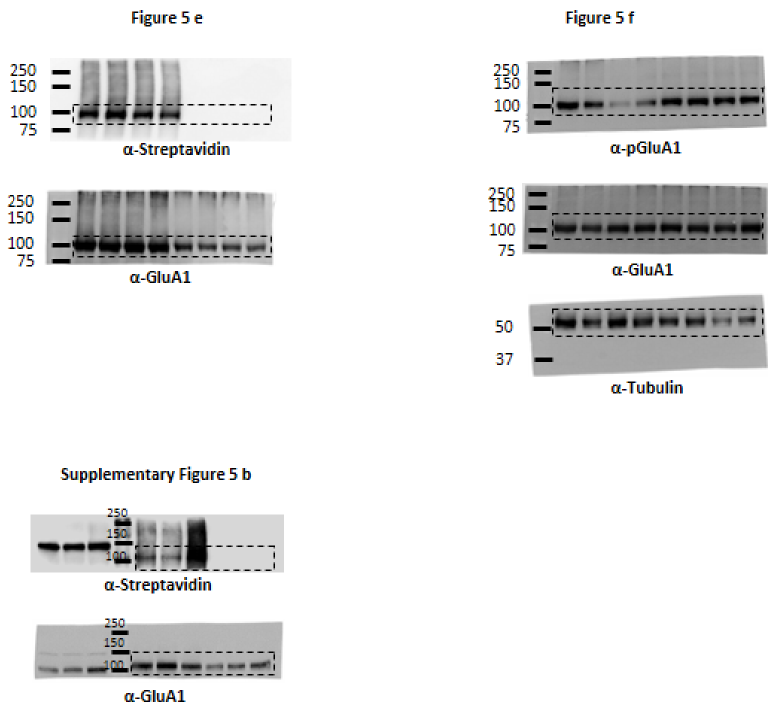

Supplementary Figure 10. The uncropped version of gel images presented in Figure 5 and Supplementary Figure 5.

Figure 6 e

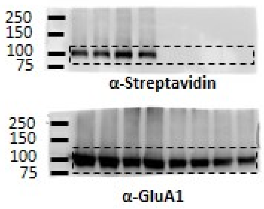

Supplementary Figure 11. The uncropped version of gel images presented in Figure 6.

Supplementary Table 1. PRIMERS

Primer sequences used for ChIP analysis.

Gene		Primer sequence
zDHHC3 pFRE1	FW	5’-TTCTGGATGCTGCTAGCTCC-3’
	RV	5’-CAAAGAGAGGCAGAGCTGC-3’
zDHHC3 pFRE2	FW	5’-AATGACTGAATTTTGGCATTCC-3’
	RV	5’-CTC AGT CCT GCT TTT GAA CG-3’
zDHHC3 pFRE3	FW	5’-TTGCTCAGCTAGAAAATTTTGG -3’
	RV	5’- GAAGAAATGACCGTGTACAGC-3’
zDHHC3 pFRE4	FW	5’-TAGCCATCACTTTTCCACTGG-3’
	RV	5’- CAAGAGACTCTGAGTTCTGC -3’

Abbreviations: zDHHC3 pFRE, FoxO3a putative Responsive Elements; FW, forward; RV, reverse

Primer sequences used for Real Time PCR analysis.

Gene		Primer sequence
zDHHC2	FW	5’-AATCCTGAAAACCACCAGTTTCC-3’
	RV	5’-GTCTCGTTCTCCATAGTTAATGC-3’
zDHHC3	FW	5’-CAGTCATCCTGCTCATCCTGC-3’
	RV	5’-CTCAGTCCTGCTTTTGAACG-3’
zDHHC4	FW	5’- GATCTCTACCAGGAAACTTACC-3’
	RV	5’-TACAGAGCAAAGCACAAGTAGC-3’
zDHHC5	FW	5’-GGTGTCTCAGAGACAGAGG-3’
	RV	5’-ATTCACACAGAAATCTCATAAGTGG-3’
zDHHC7	FW	5’-ATGTACATAGCTCTGTCTTCG-3’
	RV	5’-CTCGCTCTTCAGCCTCTCG-3’
zDHHC8	FW	5’-CTTCTATTCATCCAGCACCTCC-3’
	RV	5’-CCTTCTTAACCAGCGTGTGC-3’
zDHHC12	FW	5’-GCTGGTAGTTCTGCTGTGG-3’
	RV	5’-GTGTTCCTGGCTACCAGG-3’
zDHHC13	FW	5’-AATCAACTCTTTCAGATTGCATTTCTGG-3’
	RV	5’-GGATGTCCAATCTATGATGCAGG-3’
zDHHC15	FW	5’-TGGTGATGGACACTCCTTCC-3’
	RV	5’-ACACAGAAAATGTGAAACCTGC-3’
zDHHC17	FW	5’-CAGCTGTACCAGATAACATGTTTAGG-3’
	RV	5’-GGTCCAGTCCACGATAACAGG-3’
zDHHC20	FW	5’-GGACAAATAACCATGTTACAGTGG-3’
	RV	5’-CTCTGCAGTGCTTCTGTGG-3’

Abbreviations: *zDHHC*, palmitoyl transferase; FW, forward; RV, reverse

Supplementary Table 2. ANTIBODIES

Primary Antibody	Host and dilution	Catalogue reference
α-AKT	Rabbit 1:1000	Cell Signaling #9272
α-pAKT Ser^{473}	Rabbit 1:1000	Cell Signaling #4060
α-GSK3-3β	Rabbit 1:1000	Cell Signaling #12456
α-phospho GSK-3β Ser^{9}	Rabbit 1:1000	Cell Signaling #9336
α-FoxO3a	Rabbit 1:1000	Cell Signaling #2497
α-phospho FoxO3a Ser^{253}	Rabbit 1:1000	Cell Signaling #9466
α-GluA1 (D4N9V)	Rabbit 1:300	Cell Signaling #13185
α-GluA1 (N-terminus)	Mouse 1:500 – 1:1000	Millipore #2263
α-PSD95 (D27E11)	Rabbit 1:500 – 1:1000	Cell signaling #3450
α-Tubulin	Mouse 1:1000	Sigma #T6074
α-MAP2	Mouse 1:500	Sigma # M9942
α-58K Golgi	Mouse 1:300	Novus Biologicals # NB600-412
Rhodamine phalloidin	1:500	Thermo Fisher #R415
α-phospho GluA1 Ser^{845} (D10G5)	Rabbit 1:1000	Cell signaling #8084
α-GluA2	Mouse 1:1000	Millipore #391
α-GluA2 Ser^{880}	Rabbit 1:1000	Millipore #07-294
α-NMDAR1	Mouse 1:500	BD Bioscience #556308
α-Actin	Rabbit 1:1000	Biorbyt #10033
α-NMDAR2A	Mouse1:1000	Santa cruz #515148
α-NMDAR2B	Mouse 1:500	BD Bioscience #610417
α-zDHHC3	Rabbit 1:1000	Abcam #31837
α-GluA3	Rabbit1:1000	Abcam #40845
α-GluA4	Rabbit 1:1000	Cell signaling #8070P
α-GluK2	Rabbit 1:1000	Abcam #124702
α-GABA A Receptor gamma 2	Rabbit 1:500	Abcam #87328
α-Biotin	Rabbit 1:1000	Novus biologicals #NBP2-15589
streptavidin-HRP	1:2500	Thermo Fisher #21126
Alexa Fluor 488 anti-rabbit	Donkey 1:1000	Thermo Fisher #A21206
Alexa Fluor 488 anti-mouse	Donkey 1:1000	Thermo Fisher #A2120
Alexa Fluor 633 anti-rabbit	Goat 1:1000	Thermo Fisher #A21072

Supplementary References

[1] Lein, E.S. et al. Genome-wide atlas of gene expression in the adult mouse brain *Nature* 445, 168-176 (2007).
